# Supplementary material for: Perioperative Hyperspectral Imaging to Assess Mastectomy Skin Flap and DIEP Flap Perfusion in Immediate Autologous Breast Reconstruction: A Pilot Study
Source: Diagnostics (Basel). 2022 Jan 13;12(1):184. doi: 10.3390/diagnostics12010184 (PMC8774932; doi:10.3390/diagnostics12010184)
Supplement: Supplementary file 1 [file diagnostics-12-00184-s001.zip › Supplementary Figures.pdf]

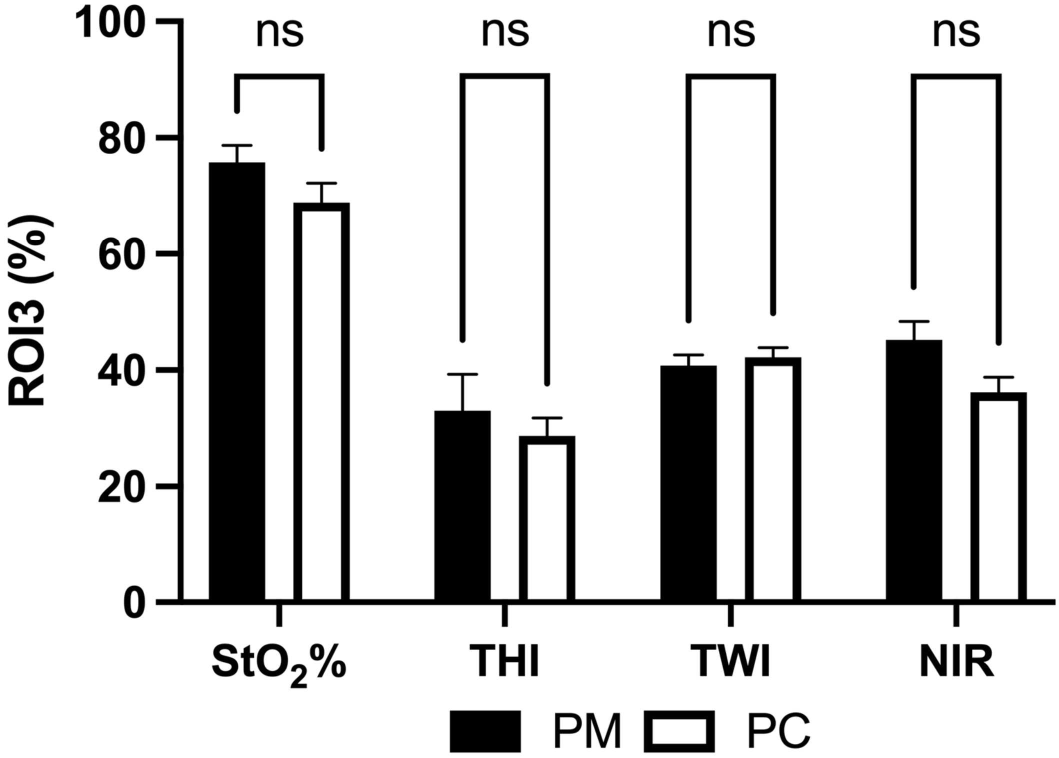

Figure S1. Comparison in perfusion parameters between the moment following mastectomy (PM = postmastectomy) and following temporarily clipping the internal mammary artery (PC = post-clip) in ROI 3 (medial side of the mastectomy skin flap). Data are expressed as mean ± SEM. Student t-test was used $p > 0.05$ was considered non-significant.

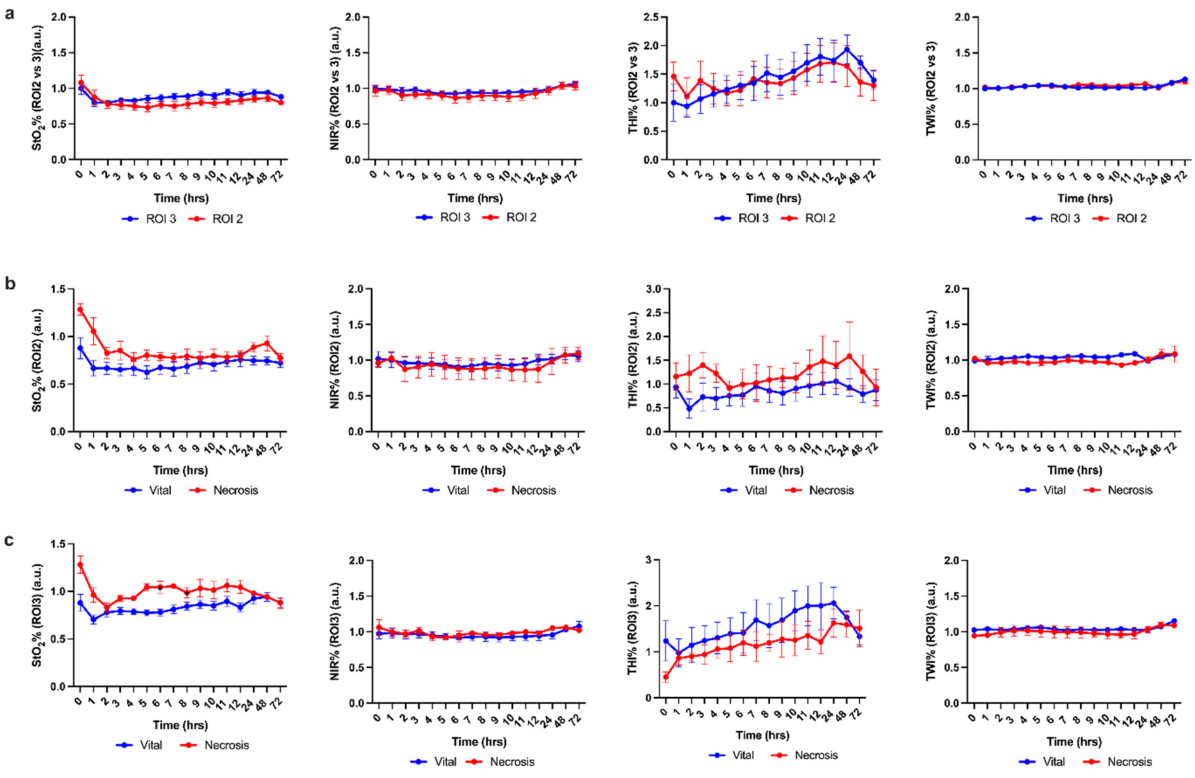

Figure S2. HSI sampling over time in (**a**) in ROI2 (red; skin island of the DIEP-flap) versus ROI3 (blue; the medial side of the mastectomy skin flap). (**b**) in the "necrosis group" versus the "vital group" in ROI2 (**c**) in the "necrosis group" versus the "vital group" in ROI3. Data are expressed as mean value. Data are expressed as mean ± SEM.
